# *Anemonia sulcata* and Its Symbiont *Symbiodinium* as a Source of Anti-Tumor and Anti-Oxidant Compounds for Colon Cancer Therapy: A Preliminary In Vitro Study

**DOI:** 10.3390/biology10020134

**Published:** 2021-02-08

**Authors:** Laura Cabeza, Mercedes Peña, Rosario Martínez, Cristina Mesas, Milagros Galisteo, Gloria Perazzoli, Jose Prados, Jesús M. Porres, Consolación Melguizo

**Affiliations:** 1 Institute of Biopathology and Regenerative Medicine (IBIMER), Center of Biomedical Research (CIBM), University of Granada, 18100 Granada, Spain; lautea@ugr.es (L.C.); mpenacontreras@ugr.es (M.P.); cristinam@correo.ugr.es (C.M.); gperazzoli@ugr.es (G.P.); melguizo@ugr.es (C.M.); 2 Department of Anatomy and Embryology, Faculty of Medicine, University of Granada, 18071 Granada, Spain; 3 Biosanitary Institute of Granada (ibs.GRANADA), SAS-University of Granada, 18014 Granada, Spain; 4 Institute of Nutrition and Food Technology (INyTA), Biomedical Research Center (CIBM), Department of Physiology, University of Granada, 18100 Granada, Spain; rosariomz@ugr.es (R.M.); jmporres@ugr.es (J.M.P.); 5 Department of Pharmacology, School of Pharmacy, University of Granada, 18071 Granada, Spain; mgalist@ugr.es

**Keywords:** *Anemonia sulcata*, *Symbiodinium*, antioxidant activity, antitumor activity, colorectal cancer

## Abstract

**Simple Summary:**

Colorectal cancer is one of the most frequent types of cancer in the population. Recently, invertebrate marine animals have been investigated for the presence of natural products which can damage tumor cells, prevent their spread to other tissues or avoid cancer develop. We analyzed the anemone *Anemonia sulcata* with and without the presence of its microalgal symbiont (*Symbiodinium*) as a source of bioactive molecules for the colorectal cancer therapy and prevention. Colon cancer tumor cells were exposed to Anemone extracts observing a remarkable cell death and a great antioxidant capacity. These preliminary results support that *Anemonia sulcata* could be a source of bioactive compounds against colorectal cancer and that the absence of its symbiont may enhance these properties. Further studies will be necessary to define the bioactive compounds of *Anemonia sulcata* and their mechanisms of action.

**Abstract:**

Recently, invertebrate marine species have been investigated for the presence of natural products with antitumor activity. We analyzed the invertebrate *Anemonia sulcata* with (W) and without (W/O) the presence of its microalgal symbiont *Symbiodinium* as a source of bioactive compounds that may be applied in the therapy and/or prevention of colorectal cancer (CRC). Animals were mechanically homogenized and subjected to ethanolic extraction. The proximate composition and fatty acid profile were determined. In addition, an in vitro digestion was performed to study the potentially dialyzable fraction. The antioxidant and antitumor activity of the samples and the digestion products were analyzed in CRC cells in vitro. Our results show a high concentration of polyunsaturated fatty acid in the anemone and a great antioxidant capacity, which demonstrated the ability to prevent cell death and a high antitumor activity of the crude homogenates against CRC cells and multicellular tumor spheroids, especially W/O symbiont. These preliminary results support that *Anemonia sulcata* could be a source of bioactive compounds with antioxidant and antitumor potential against CRC and that the absence of its symbiont may enhance these properties. Further studies will be necessary to define the bioactive compounds of *Anemonia sulcata* and their mechanisms of action.

## 1. Introduction

Marine ecosystems, the world’s main reservoir of biodiversity and a relevant source of marine natural products (MNPs), are providing a large number of compounds with antitumor, antibacterial, antiviral and anti-inflammatory properties, among others [1,2,3,4,5]. Specifically, some marine organisms such as algae and animals are a source of products with anticancer activity which have been tested as a new or adjuvant treatment [6,7,8]. In this context, advanced colorectal cancer (CRC) lacks therapeutic options, requiring new strategies and drugs to improve its prognosis. CRC, the second most lethal cancer worldwide [9], is currently treated with conventional chemotherapy, which shows low tumor specificity and high toxicity in healthy tissues. In addition, the frequent development of multidrug resistance (MDR) phenotypes reduces the effectiveness of this treatment. Accordingly, MNPs may play a fundamental role in the discovery of new drugs to improve the prognosis of advanced CRC patients [10].

In this regard, research in the marine world has provided new chemical structures with potent antitumor efficacy, but whose mechanisms of action and molecular targets remain unknown in most cases [11,12,13,14]. In fact, eight MNPs have been approved for clinical use (five as anti-tumor drugs), and 38 are being tested in clinical trials [11]. In addition, marine organisms have provided components with antioxidant activity (e.g., terpenoids, tannins, ageloline A, flavonoids, alkaloids and saponins) [12,15] which showed positive effects in cancer prevention [16,17,18,19]. Within marine invertebrates, the cnidarians (such as jellyfish, sea anemones and corals) have attracted increasing research interest in recent years [20,21,22]. *Anemonia sulcata*, a cnidarian (phylum Cnidaria) of the Actiniidae family, widespread in the Mediterranean and eastern Atlantic, produces various secondary metabolites and toxins to capture prey and defend itself from predation [23,24]. Although most of these molecules have been poorly described biologically, it is known that the main toxins act as blockers of voltage-dependent sodium and potassium channels [25,26]. *Anemonia sulcata* specimens have a symbiotic relationship with photosynthetic dinoflagellate microalgae of the genus *Symbiodinium* commonly known as zooxanthellae [27], which gives them a great competitive advantage. This association can be interrupted by a process called “bleaching”, which results from exposure of these organisms to adverse conditions such as high temperatures, high light intensity or darkness. Bleaching leads to several changes at a physiological level that may alter the generation of metabolites [28]. Previous studies have described anti-tumor and anti-angiogenic activity in extracts from anemones and the presence of antioxidant enzymes involved in the maintenance of the symbiotic relationship. In fact, three of four protein fractions with low molecular weight obtained from *Anemonia viridis* showed cytotoxic activity in several human tumor cell lines (PC3, PLC/PRF/5 and A375) [29]. Moreover, a protein fraction from *Anemonia viridis* at a very low concentration (14 nM) demonstrated anti-angiogenic activity in Human Microvascular Endothelial Cells (HMEC). The active principle responsible for this activity was a Kuntiz type inhibitor [30]. On the other hand, aqueous extracts of *Actinica equina* and *Anemonia sulcata*, which contained the alkaloid homarine, demonstrated antiproliferative activity in the RAW-264.7 cell line (murine macrophage) and human gastric adenocarcinoma cells via the activation of caspase-3 [24]. Besides, *Anemonia viridis* showed significant antioxidant activity through the presence of glutathione peroxidase and catalase in high concentrations [31,32]. 

The aim of our study was to analyze the invertebrate *Anemonia sulcata* with (W) and without (W/O) its microalgal symbiont *Symbiodinium* to determine if the bleaching process induces physiological changes that intensify the production of bioactive compounds with antitumor and antioxidant properties. These metabolites could be applied as a new strategy in the treatment and/or prevention of CRC to improve the prognosis of these patients.

## 2. Materials and Methods

### 2.1. Chemicals, Cell Lines and Animals

The trichloroacetic acid, NaOH, Na_2_CO_3_, H_2_O_2_, 3-(4,5-Dimethylthiazol-2-yl)-2,5-Diphenyltetrazolium Bromide (MTT), dimethyl sulfoxide (DMSO), pepsin, pancreatin, proteases from *Bacillus licheniformis* and *Aspergillus oryzae*, bile salts, gallic acid, Folin–Ciocalteu reagent, Coomassie brilliant blue R250, Bradford Reagent and 2,2′-Azino-bis(3-ethylbenzothiazoline-6-sulfonic acid) (ABTS) were purchased from Sigma-Aldrich; Thermo Scientific™ PageRuler™ Plus Prestained Protein Ladder from Thermo Fisher Scientific; absolute ethanol and HCl from Panreac; PI/RNase solution and Annexin V FITC apoptosis detection kit from Immunostep; Cell Counting Kit-8 (CCK-8) from Dojindo Laboratories; and Biotech RC Dialysis Membranes from Repligen.

The cell lines used from human colorectal cancer T84, SW480, HT29 and HCT15 were purchased from the American Type Culture Collection (ATCC, USA). The human no-tumor colon cell line CCD18 was obtained from the Centre for Scientific Instrumentation of the University of Granada (CIC-UGR, Spain). The murine cell line of colorectal cancer MC38 was kindly provided by Dr. J. Scholl (Public Health Service, National Institutes of Health, Bethesda, MD, USA). All the cell lines were cultured with Dulbecco’s Modified Eagle’s Medium (DMEM) (Sigma-Aldrich, St. Louis, MO, USA) supplemented with 1% of an antibiotics mixture (penicillin = 10.000 U/mL and streptomycin = 10 mg/mL) (Sigma-Aldrich) and with 10% heat-inactivated fetal bovine serum (Thermo Fisher Scientific, Waltham, MA, USA) in a humidified atmosphere at 37 °C and 5% CO_2_. The *Anemonia sulcata*, animals with (W) and without (W/O) its microalgal symbiont *Symbiodinium*, were obtained from the company iMare Natural S.L. 

### 2.2. Proximate Composition and Fatty Acid Profile

Four lyophilized specimens were used for the proximal composition analyses. Moisture content analysis were performed by the calculation of weight loss in the lyophilization process. Incineration in an oven at 550 °C was carried out for the determination of ashes by gravimetry as a measure of total mineral content. The protein content was analyzed by Kjeldahl’s method, using 6.25 as a conversion factor. In addition, fat analysis was made by Soxhlet method after an acid hydrolysis. Finally, the fatty acid profile was analyzed by gas chromatography following the protocol of Martínez et al., [33].

### 2.3. Anemonia sulcata Crude Extract Preparation W and W/O the Symbiont

Two complete frozen animals (2 × W and 2 × W/O) were thawed, lightly washed with water, weighed (~18–18.5 g) and cut with scissors. After that, animals were mechanically homogenized with a high-speed homogenizer (IKA T10 Basic ULTRA-TURRAX9) followed by sonication at 50% power for 1 min in cycles of 30 s in a phosphate buffer (50 mM and pH 7.2). This process was done at 4 °C. A volume of 20 mL was reserved for in vitro digestion (see below). Another volume of 10 mL was centrifuged 12,000 rpm for 15 min at 4 °C to remove tissue debris. After the centrifugation the supernatant was collected and used for in vitro studies with cultured cells.

### 2.4. Ethanolic Extraction of Anemonia sulcata W and W/O the Symbiont

Four complete frozen animals (4 × W and 4 × W/O) were lyophilized and 0.5 g per replicate of the lyophilized samples was used to make the ethanolic extraction. A modified methodology of Kapravelou et al. was used [34]. A volume of 15 mL of a solution of absolute ethanol, 12 N HCl and double-distilled water (50:0.25:50) was added to the sample and the mixture was stirred and sonicated 4 to 5 cycles of 10 s. Nitrogen was then bubbled into the samples to avoid oxidation, and, at pH 2, the samples were extracted for 30 min. After that, the samples were centrifuged (3500 rpm/10 min) and the supernatant collected. With the pellet the complete extraction was made again in 10 mL of the previous solution. All the processes were made at 4 °C. An aliquot of the supernatant was kept at −20 °C for antioxidant experiments and other part was evaporated under vacuum to eliminate the ethanol and tolerate its use in cultured cells. 

### 2.5. Anemone Protein Hydrolysates

The anemone protein hydrolysate (PH) was prepared from crude homogenates by an alkaline water extraction and hydrolysis process with protease treatment. A volume of 40 mL of distilled water was added to 9 mL of sample and heated to 33 °C under continuous stirring for 30 min. The pH was adjusted to 8.8 with 3N KOH. The sample was centrifuged 7000 rpm for 5 min. The supernatant was reserved and the pellet was used to repeat the extraction. The two supernatants obtained were mixed and heated to 47 °C for 20 min under continuous stirring and 500 µL of CaCl_2_ and MgSO_4_ 100 mM were added and the mixture incubated at 47 °C for 20 min. After that, enzymatic digestion began with the addition of protease from *Bacillus licheniformis* (0.3 AU/g protein) at 47 °C and pH 8.8 for 30 min. Then, protease from *Aspergillus oryzae* was added (100 AU/g protein) using the same conditions. After the hydrolysis process, the sample was lyophilized and prior to be added to cell cultures, the lyophilized extract was resuspended in double-distilled water and heated 10 min at 95 °C to inactivate the proteolytic enzymes. 

To ensure that protease treatment was successful, the samples were analyzed by sodium dodecyl sulfate–polyacrylamide gel electrophoresis (SDS-PAGE) and Coomassie brilliant blue staining. Briefly, the protein content of the samples was measured by Bradford assay and equal amounts of protein (35 µg) were loaded in each line of the gel (final concentration of acrylamide in the running gel: 15%). Crude homogenates were used as control. Molecular weight markers from 10 to 250 kDa were used. Lastly, gels were fixed and stained with Coomassie brilliant blue R250. 

### 2.6. Mass Spectrophotometry Analysis 

A volume of 10 µL of crude homogenates (HOMG) W and HOMG W/O was used for the mass spectrophotometry analysis that was made with ultra-performance liquid chromatography (UPLC) (Acquity HClass, Waters) coupled to a quadrupole-time-of-flight (QTOF) mass spectrometer (Synap G2, Waters). The chromatographic conditions in [33] were used. The chromatograms were analyzed with MassLynx V4.1 software to tentatively identify compounds in the crude homogenates based on their mass fragments (MS) and retention times (RT) by the use of the Chemspider database. 

### 2.7. In Vitro Digestibility of the Crude Extracts

The in vitro digestibility of crude homogenates W and W/O symbiont was done according to Porres et al. (2005) with minor modifications [35]. Briefly, a volume of 36 mL of 0.01 N HCl was mixed with 1.9 mL of each sample and 0.1 N HCL was further added to the mixture until pH 2 was reached. For the simulated gastric digestion, 10 mL of the former mixture were mixed with 10 mL of 0.01 N HCl and 1 mL of 0.16 g/mL (0.1 N HCl) pepsin and incubated in a shaking water bath at 37 °C for 2 h. Negative controls were made with the same volume of 0.01 N HCl instead of the sample. Prior to the simulated intestinal digestion, a pH compensation step of 30 min was performed with 0.1 N NaHCO_3_ added into the dialysis bags which were placed in the digestion vessels in a shaking water bath at 37 °C. Then, 5 mL of a 0.1 N NaHCO_3_ solution with pancreatin (4 mg/mL) and bile salts (25 mg/mL) were added, and the was mixture incubated in a shaking water bath at 37 °C for 2 h. Once digestion was finished, the contents inside (sample dialyzed and potentially absorbable) and outside (sample retained that could reach the colon) the dialysis bags were collected and kept at −20 °C to be used in the following determinations.

### 2.8. Antioxidant Activity of the Samples

#### 2.8.1. Quantification of Total Polyphenols

This assay was performed by the use of a modified Folin–Ciocalteu colorimetric assay [36]. A volume of 125 µL of diluted samples from crude homogenates, ethanolic extracts, protein hydrolysates, the in vitro digestion products or the standard solution of gallic acid (0–600 mg/L) was mixed with 500 µL of double-distilled water and 125 µL of Folin–Ciocalteu reagent. After 6 min of incubation, a solution of 1.25 mL of a 10% (*w*/*v*) Na_2_CO_3_/1 M NaOH was added, and the volume was made up with water to 3 mL and incubated for 90 min. Then, after centrifugation (2000 rpm/2 min), the optical density of the sample supernatant was measured at 750 nm (Multiskan™ FC Microplate Photometer, Thermo Fisher Scientific). The results were expressed as μg of gallic acid equivalent (GAE) per mg of sample.

#### 2.8.2. Free Radicals’ Uptake/Retention (ABTS)

This assay was performed based on the method of Miller et al. (1993), who used 2,2′-Azino-bis(3-ethylbenzothiazoline-6-sulfonic acid) (ABTS) to measure the total antioxidant capacity of a fluid [37]. A volume of 6 µL of the samples of crude homogenates, ethanolic extracts, protein hydrolysates and the in vitro digestion products or standard solution of gallic acid (0–60 mg/L) was mixed with 294 µL of ABTS and incubated for 5 min. After that, the optical density of the samples was measured at 620 nm (Multiskan™ FC, Microplate Photometer, Thermo Fisher Scientific). The blank was made with 6 µL of water and 294 µL of ABTS. The results were expressed as μg of gallic acid equivalent (GAE) per mg of sample.

#### 2.8.3. Antioxidant Activity in Cultured Cells

To test the antioxidant activity in cultured cells, human colorectal cancer HT29 cell line was used. Cells were seeded in 96-well plates at a density of 5 × 10^4^ cells/well in 150 µL of supplemented DMEM. After 24 h, the culture medium was replaced with serum-free DMEM. One day later, the treatments with crude homogenates, ethanolic extracts and protein hydrolysates were added at non-toxic concentrations of 0.5 and 0.05 µg/mL for 24 h. Then, the medium that contained treatments was discarded, new fresh serum-free medium was added and some wells were treated with H_2_O_2_ in concentrations of 2.7 and 3 mM. After 6 h, the medium was replaced again for fresh serum-free medium for 12 h. Then, the MTT (3-(4,5-Dimethylthiazol-2-yl)-2,5-Diphenyltetrazolium Bromide) protocol was carried out. Briefly, 30 µL of MTT was added per well for 4 h in culture conditions. Then, the medium was discarded, and 200 μL of dimethyl sulfoxide (DMSO) plus 25 μL of Sorensen’s glycine buffer (glycine 0.1M, NaCl 0.1M, pH:10.5 with 0.1 NaOH) were added per well to dissolve formazan crystals. After 5 min of incubation at room temperature, the optical density of the wells was measured at 570 nm and a reference wavelength of 690 nm (Titertekmultiscan Colorimeter, Flow, Irvine) to determine the relative proliferation (%RP) of treated cells. 

### 2.9. Antiproliferative Activity in CRC Cells

The cell lines were seeded in 48-well plates at a density of 5 × 10^3^ cells/well for T84, CCD18 and SW480 and HCT15, 15 × 10^3^ cells/well for HT29 3 × 10^3^ cells/well for MC38 in 300 μL of supplemented DMEM. The next day, treatments of crude homogenates (HOMG), heat-treated HOMG exposed to 96 °C/5 min in a thermoblock or 56 °C/30 min in a water bath, HOMG after 1–4 freeze–thaw cycles, two replicates of ethanolic extracts (ETOH), the PH and the digestion products from *Anemonia sulcata* W and W/O symbiont were added in increasing concentrations. Negative control cells were untreated cells. After 72 h, the MTT assay was performed to calculate the percentage of relative proliferation (%RP) of cultures.

### 2.10. Cell Cycle and Apoptosis Assay

Cell lines were seeded in 6-well plates at a density in cells/well of 7.5 × 10^4^ for MC38 and SW480, 1 × 10^5^ for T84 and CCD18 and 1.5 × 10^5^ for HT29 and HCT15 in 2 mL of supplemented DMEM. The next day, the medium was discarded and replaced for the same quantity of serum-free DMEM to induce cell cycle arrest. After 24 h, the medium was replaced again for supplemented DMEM and treatments with HOMG W and W/O symbiont at half-maximal inhibitory concentration (IC_50_) for 48 h and the same treatments heated (95 °C/5 min). After the incubation time elapsed, cells were detached, centrifuged (3000 rpm, 5 min) and, for the cell cycle assay, fixed with 70% ethanol–PBS (900 µL) for 30 min at 4 °C. After centrifugation, 500 µL of PI/RNase solution were added to cells for 15 min at room temperature. For annexin V/PI staining, cells were washed with PBS and resuspended in 1× annexin binding buffer (1 × 10^6^ cells/mL) with 5 µL of annexin V-FITC and PI according to manufacturer’s instructions. After 15 min of incubation at room temperature in the darkness, 400 µL of 1× annexin binding buffer were added. Finally, all samples were analyzed with FACScan (Becton Dickinson).

### 2.11. Wound Healing Assay

The cell line T84 was seeded in 12-well plates with 3 × 10^5^ cells/well in 1 mL of culture medium. The next day the wound was made with a 100 µL pipette tip followed by washing with PBS to remove the detached cells and adding serum-free medium (1 mL). Then, treatments with crude homogenates at a non-toxic dose, IC_10_ and IC_30_ were added for 72 h. Every day, images were taken with an inverted light microscope Olympus CKX41 (Olympus Corporation) to observe the evolution of the wound closure. Cell migration was measured with MRI_Wound_Healing_Tool of ImageJ software and relative wound area (%) was determined in relation to 0 h of exposition. 

### 2.12. Multicellular Tumor Spheroids Antitumor Assay

To generate multicellular tumor spheroids (MTS), 50 µL of 1% agarose (*w*/*v*) were added to 96-well plates. Once agarose was gelled, 250 MC38 cells/well were seeded in 200 µL of supplemented DMEM and the plate was centrifuged (800× *g*/5 min). After 72 h, the MTS were formed and in this moment were treated with HOMG W and W/O symbiont at IC_50_, 2 × IC_50_ and 4 × IC_50_ doses for 3 days. Every day from the beginning of treatment, MTS volume was measured by light microscope images analyzed with ImageJ software. At the end of the exposure time cell proliferation was tested with the Cell Counting Kit-8 (CCK-8). Briefly, CCK-8 was added to each well to reach a final concentration of 10%. After 4 h of incubation, optical density of the wells was measured at 450 nm and a reference wavelength of 620 nm (Titertekmultiscan Colorimeter, Flow, Irvine) to determine the relative proliferation (%RP) of MTS.

### 2.13. Statistical Analysis

All results were expressed as the mean ± standard deviation (SD). Statistical analysis was performed by the Student’s t-test and one-way ANOVA (SPSS v.15, SPSS, Chicago, USA). Values of *p* < 0.05 were considered significant.

## 3. Results and Discussion

### 3.1. Proximate Composition and Fatty Acid Profile

**Figure 1:**
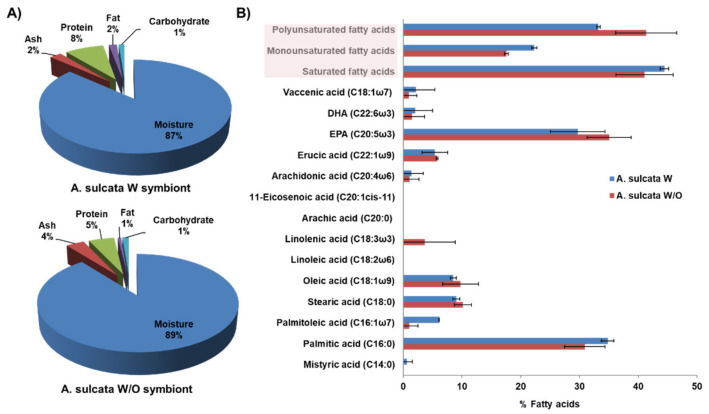
Composition of *Anemonia sulcata* with (W) and without (W/O) its microalgal symbiont *Symbiodinium*. (**A**) Graphical representation of the proximate composition. (**B**) Graphical representation of the fatty acids. The sum of saturated, monounsaturated and polyunsaturated fatty acids is highlighted in the pink box. DHA and EPA are cis-4,7,10,13,16,19-docosahexaenoic acid and 5,8,11,14,17-eicosapentaenoic acid, respectively. Data are represented as the mean ± standard deviation of duplicate samples.

Proximate composition analysis revealed a high percentage of water in the anemone, both with (W) and without (W/O) its microalgal symbiont (87% and 89%, respectively). In addition, a high content of protein (8% and 5%, respectively) (Figure 1A), polyunsaturated (PUFA) and saturated fatty acids (SFA) (Figure 1B) was detected in both samples. However, *Anemonia sulcata* W/O its symbiont showed a higher percentage of PUFA (41.4%) than *Anemonia sulcata* W its symbiont (33.2%). The PUFA with the highest concentration was 5,8,11,14,17-eicosapentaenoic acid (EPA), which also showed higher levels in *Anemonia sulcata* W/O its symbiont (35.1% W/O versus 29.7% W). Furthermore, the presence of oleic acid was similar in both samples (9.8% W/O and 8.5% W) and the concentrations of arachidonic acid were almost undetectable. Fatty acid analysis suggested an added nutritional value of *Anemonia sulcata* W/O its symbiont based on its higher percentage of PUFA. In addition, the presence in both samples (especially in W/O) of EPA, a fatty acid with protective activity against CRC [38], could warrant its preventive application in this pathology. In fact, the presence of PUFA (n-6 PUFA, EPA and DHA) in the diet has been correlated with a significant decrease in digestive cancer risk, which may be regulated by the ingestion of vegetables, fruits, vitamin C and fiber [39]. Moreover, PUFAs not only have a protective effect in CRC but also improve its treatment by increasing the activity of antioxidant enzymes, reducing lipid peroxidation and inhibiting harmful derivatives of arachidonic acid, thus warranting their use as adjuvants in radio and chemotherapy [40]. In relation to this last point, the absence of arachidonic acid—which is associated with an increased risk of CRC [38]—in the anemones, both with (W) and without (W/O) its microalgal symbiont, reinforces our hypothesis.

### 3.2. Anemone Protein Hydrolysates

**Figure 2:**
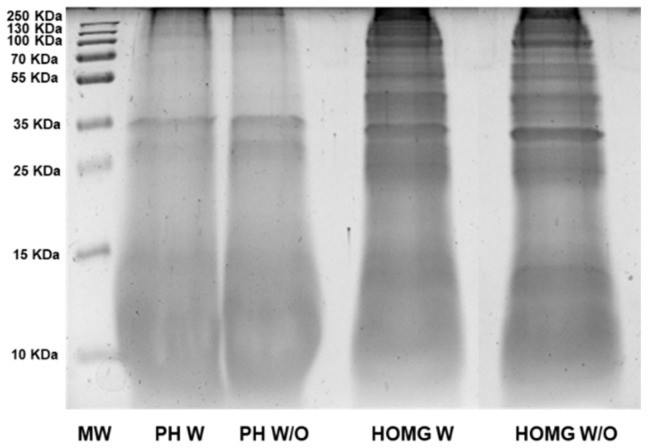
SDS-PAGE of proteins from *Anemonia sulcata* with (W) and without (W/O) its microalgal symbiont *Symbiodinium*. The protein hydrolysates were compared to the crude homogenates to observe different patterns of proteins, indicating that the enzymatic digestion had been carried out correctly. Molecular weight (MW); protein hydrolysates (PH); crude homogenates (HOMG).

SDS-PAGE was performed to ensure that the enzymatic digestion was successful and to observe differences in the pattern of proteins between the crude homogenates. This procedure caused changes in the pattern of proteins in the PHs compared to the crude homogenates (Figure 2). In the case of PHs, a reduction in the number of main protein bands and in their density compared to the crude homogenates was observed, indicating proteolysis of large proteins. This reduction was similar in the samples W and W/O symbiont.

### 3.3. Mass Spectrophotometry Analysis

**Figure 3:**
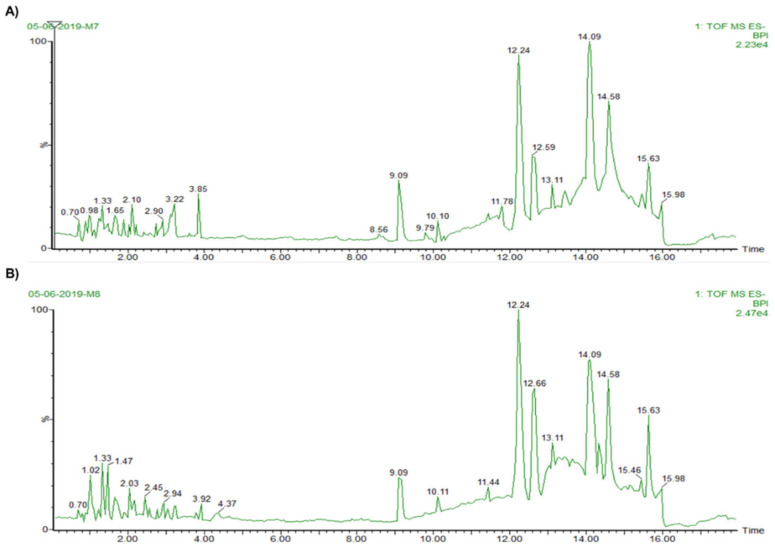
Chromatograms of the crude homogenates from *Anemonia sulcata* with (**A**) and without (**B**) its symbiont.

Chromatograms from the mass spectrophotometry analysis of the crude homogenates of *Anemonia sulcata* (Figure 3) were analyzed to detect possible bioactive substances. From the tentative compounds (Table 1), gadusol; neogambogic acid; casearupestrin A and B; olivoretin A, B, C and E; and ophioxanthol were present in both HOMG W and W/O symbiont. Interestingly, gadusol and ophioxanthol have been previously described in other marine organisms. Gadusol blocks UV radiation, showing photoprotective and antioxidant activities [41]. Ophioxanthin is a carotenoid sulfate from *Ophioderma longicaudum* [42]. The carotenoid family has widely demonstrated antioxidant activity as a scavenger of ROX, O_2_ and peroxyl radicals, as well as antitumor activity and protective effects (cancer chemoprevention) in cancer development [43]. Furthermore, 1-arachidonoylglycerone 3-phosphate was detected in HOMG W, although no function has been previously described for this compound. The presence of some of these compounds in the crude homogenates from *Anemonia sulcata* could be the cause of the observed activities that will be discussed in the following sections.

### 3.4. In Vitro Digestibility

After simulated gastric and intestinal digestion and intestinal absorption, the bioaccessible or dialyzed (DIAL) amount of HOMG W and HOMG W/O was 1 ± 0.27 and 1.17 ± 0.42 mg of dialyzed fraction/mL sample, respectively, while the fraction of the digested sample potentially dialyzable (100%) was 1.5 and 1.75 mg/mL, respectively. Thus, the percent dialyzed from the sample was 64.01 ± 17.2% and 66.58 ± 23.8% for HOMG W and HOMG W/O, respectively. Our results indicate that more than a half of the anemone digested in both samples was potentially bioavailable, suggesting that they could reach the tissues via the bloodstream and exert their antitumor and/or antioxidant effects [50,51]. However, non-dialyzable digestion products could conserve these activities after digestion and exert their effects in the colon.

### 3.5. Antioxidant Activity of the Samples

#### 3.5.1. Quantification of Total Polyphenols

Total polyphenols content was measured in the crude homogenates from *Anemonia sulcata* and their digestion products. Ethanolic extracts and protein hydrolysates were obtained (see Section 2.4 and Section 2.5) to carry out the same test. Total polyphenols content was similar in the crude homogenates W and W/O symbiont (Table 2). The simulated digestion process decreased this polyphenols content in both samples, although the reduction was lower in HOMG W/O DIAL. This finding is in agreement with previous observations in extracts from brown seaweed (rich in phlorotannin), in which reduction in total polyphenols was observed after a simulated digestion [52]. Microencapsulation of the extracts has been used to overcome this limitation because it allows protecting them from the acidic environment and enzymatic activity of digestion, preserving its antioxidant activity [53]. On the other hand, the ethanolic extracts and protein hydrolysates only showed a slight decrease in the quantity of polyphenols compared to the crude homogenates. In fact, ethanolic extracts with the symbiont showed similar values to the crude homogenates (12.55 and 13.37 µg GAE/mg sample for EXTOH W and HOMG W, respectively). Therefore, none of the extraction processes reduced the quantity of total polyphenols of the original specimen.

#### 3.5.2. Assessment of Free Radicals’ Uptake/Retention

The uptake/retention of free radicals by the crude homogenates from *Anemonia sulcata* and their digestion products, ethanolic extracts and protein hydrolysates was analyzed using ABTS. As shown in Table 3, the crude homogenates W and W/O the symbiont showed higher antioxidant capacity than their digestion products. In fact, Sun et al. (2019) described that pH conditions and digestive enzymatic activity may reduce the ability of ABTS radical scavenging of some substances [54]. Interestingly, samples of crude homogenates W/O the symbiont and their dialyzed digestion products showed significantly higher capacity of uptake/retention of free radicals (*p* < 0.05). Conversely, the retained samples of the HOM W symbiont showed a ~2-fold higher antioxidant capacity vs. W/O samples, although no significant differences were found. These results suggest that oral administration of HOMG W/O could be useful due to its antioxidant activity after digestion and dialyzability. However, the conserved antioxidant activity of the retained fraction could be modulated by other factors such as the intestinal microbiota [55]. Finally, analysis of the ethanolic extracts and protein hydrolysates showed that EXTOH W/O preserved the antioxidant activity, whereas the EXTOH W reduced this activity in comparison with the crude homogenates. In addition, the protein hydrolysates W showed a greater antioxidant capacity (with a higher percent inhibition) compared to the rest of the extracts, and, therefore, a greater capacity for reactive oxygen species (ROS) uptake.

#### 3.5.3. Antioxidant Activity in Cultured Cells

**Figure 4:**
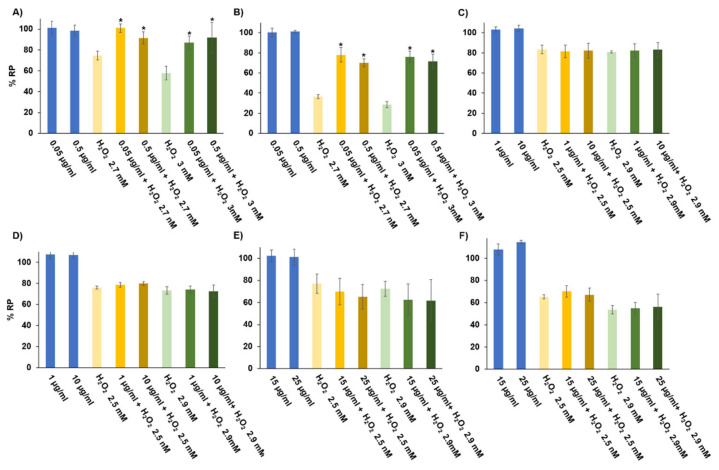
Antioxidant activity of *Anemonia sulcata* in cultured cells. The HT29 human CRC cell line was exposed to H_2_O_2_ and non-toxic doses of: crude homogenates with symbiont (W) (**A**); crude homogenates without symbiont W/O (**B**); ethanolic extract W (**C**); ethanolic extract W/O (**D**); protein hydrolysate W (**E**); and protein hydrolysate W/O (**F**). Relative proliferation was obtained by the MTT protocol. Data are represented as the mean ± standard deviation of octuplicate cultures. The symbol * indicates significant differences with H_2_O_2_ treatment.

It is known that cnidarians, including *Anemonia sulcata*, produce high quantities of antioxidant enzymes (e.g., glutathione peroxidases) to eliminate ROS generated by their symbionts [32]. Moreover, the microalga *Symbiodinium* produces ROS in normal conditions. However, high temperatures, high solar illumination or light depletion may affect the symbiotic relationship [56,57], increasing ROS production and inducing overexpression of antioxidant mechanisms to survive. This situation is known as “coral bleaching”. For example, a decrease in *Symbiodinium* photosynthesis (30–50%) together with a reduction in superoxide dismutase activity and antioxidant activity of peridinin, diatoxanthin and the ubiquitin–proteasome pathway have been observed after bleaching [57,58]. In our case, it is possible that the light stress situation that was induced to inactivate the symbiont of *Anemonia sulcata* was able to trigger a greater production of ROS and, consequently, of antioxidant molecules. To determine the modulation of antioxidant activity by the crude homogenates, ethanolic extracts and protein hydrolysates from *Anemonia sulcata*, an assay with non-toxic doses in HT29 cells was carried out. H_2_O_2_ exposure was used as a positive control. As shown in Figure 4, both HOMG W and HOMG W/O induced a clear protective effect. In fact, all doses of HOMG W significantly increased the proliferation rate of HT29 cells (*p* < 0.05), highlighting the change observed between H_2_O_2_ 3 mM (57.89%) and H_2_O_2_ 3 mM + HOMG W at 0.05 and 0.5 µg/mL (86.88% and 91.81%, respectively). Better results were obtained with HOMG W/O, which also increased this percentage between H_2_O_2_ 3 mM (28.46%) and H_2_O_2_ 3 mM + HOMG W/O at 0.05 and 0.5 µg/mL (75.97% and 71.49%, respectively). Thus, the antioxidant protective capacity was higher with the HOMG W/O pre-treatment (%RP increase up to 2.7 times) than with the HOMG W pre-treatment (%RP increase up to 1.6 times). These results suggest that the greater antioxidant activity of the HOMG W/O compared to the HOMG W/O is related to the light stress phenomenon (“coral bleaching”). Finally, treatment with ethanolic extracts and protein hydrolysates did not induce any significant protective effect in HT29 cells.

### 3.6. Antitumor Activity

#### 3.6.1. Antiproliferative Effect in Cultured Cells

**Figure 5:**
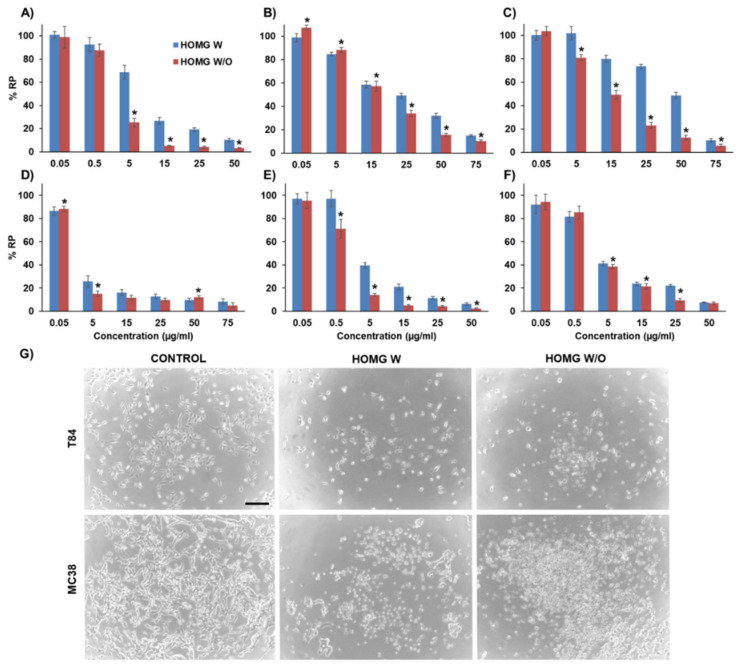
Antiproliferative activity of the crude homogenates in colon cells. T84 (**A**), HT29 (**B**), HCT15 (**C**) and SW480 (**D**) human colon cancer cells; MC38 murine colon cancer cell line (**E**); and CCD18 non-malignant human colon cell line (**F**) were exposed to the crude homogenates of *Anemonia sulcata* with (W) and without (W/O) the microalgal symbiont *Symbiodinium* for 72 h. Relative proliferation is expressed as %RP. Data are represented as the mean ± standard deviation of triplicate cultures. The symbol * indicates significant differences between crude homogenates with (W) and without (W/O) symbiont. Microscopy images of T84 and MC38 colon cancer cells treated with 25 µg/mL of crude homogenates for 48 h (**G**). Control cells represent the untreated cells. The images were taken at 4× magnification (scale bar = 200 µm).

Colon cells exposed to the crude homogenates showed that HOMG W/O caused a significantly higher cell death rate than HOMG W (*p* < 0.05). As shown in Figure 5, the HOMG W and W/O IC_50_ values were 7.78 and 2.36 µg/mL for T84, 17.84 and 19.86 µg/mL for HT29, 40.76 and 13.02 µg/mL for HCT15 and 3.45 and 1.21 µg/mL for MC38, respectively. Minor IC_50_ differences were detected in CCD18 cells (3.58 and 2.81 µg/mL, respectively). Only the SW480 colon cancer cell line did not show significant differences between the two treatments (0.54 µg/mL W and 1.09 µg/mL W/O), although it was the most sensitive cell line to both treatments. In addition, the antiproliferative activity of both extracts was modulated by temperature. In fact, the crude homogenates exposed to high temperatures (95 and 56 °C) or freeze–thaw cycles decreased their antitumor effect (Figure S3A,B). In the case of freeze–thaw cycles, this change correlated with the number of cycles, with loss of effect after four cycles (Figure S3C,D). These results suggest that those substances responsible for the antiproliferative activity, such as proteins, could be sensitive to temperature increase. Indeed, low concentrations (14 nM) of the small protein BDS-5 obtained from *Anemonia viridis* can inhibit endothelial cell growth and angiogenesis [30]. Furthermore, a fraction (Fraction II) of low molecular weight proteins from *Anemonia viridis* inhibited the growth of A375, PLC/PRF/5 and PC3 cancer cells by apoptosis without affecting ROS or caspases [29]. An increase in protein content has been observed in samples from *Entacmaea quadricolor* (i.e., venom) after the bleaching process, with an increase in nematocysts production and in toxicity [59]. Similar changes could explain the different cell toxicity between HOMG W/O and HOMG W. On the other hand, we tested the antiproliferative activity of the ethanolic extracts and protein hydrolysates from *Anemonia sulcata* W and W/O the symbiont. Interestingly, both samples showed a significant loss of antiproliferative effect in all colon cell lines (Figures S1 and S2 and Table S1). Finally, digestion products were also tested in tumor cells and showed no cell death with any treatment (Figure S4), indicating that the antitumor potential of the crude homogenates was not preserved after our simulated digestion.

#### 3.6.2. Cell Cycle Assay

**Figure 6:**
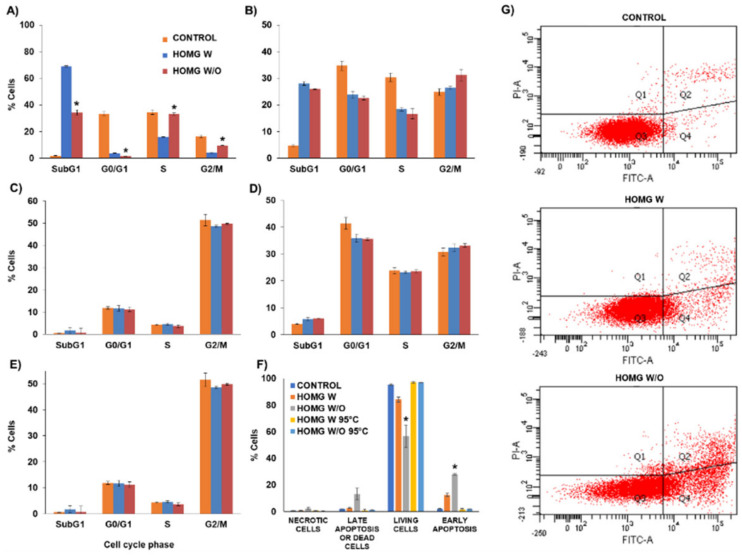
Modulation of cell cycle by the crude homogenates (HOMG) and cell death mechanism. (**A**) HT29; (**B**) HCT15; (**C**) SW480; (**D**) MC38; (**E**) CCD18; and (**F**) T84 cells were treated with HOMG W and W/O symbiont at a dose equivalent to the IC_50_ value during 48 h. Non-treated cells were used as negative controls. Results are expressed as the percentage of labeled cells in each cell cycle phase or type of cell death. (**G**) Images of flow cytometry results of T84 cells exposed to PI and annexin-V (Fluorescein, FITC). Data are represented as the mean value ± standard deviation of duplicate cultures. The symbol * indicates significant differences between crude homogenates with (W) and without (W/O) symbiont.

The cell cycle assay revealed a decrease in the cell percentage in the G0/G1 phase with an increase in the G2/M and subG1 phases in some cell lines (HT29, HCT16 and MC38) compared to untreated cells (Figure 6A–E). Specifically, the reduction in the cell percentage in the G0/G1 phase was 10.85% and 12.1% in HCT15 and 5.5% and 5.9% in MC38, while the increase in the cell percentage in the subG1 phase was 23.3% and 21.3% in HCT15 and 1.9% in MC38 for HOMG W and W/O, respectively, with no significant differences between the crude homogenates. With the annexin V staining (Figure 6F,G), the crude homogenates reduced the percentage of living cells up to 84.19% and 56.47% for HOMG W and W/O, respectively, compared to control cells (95.4%). Moreover, a significant increase (*p* < 0.05) in the number of cells in early apoptosis was observed (12.45%, 28.01% and 1.96% for HOMG W, W/O and control, respectively) with a smaller increase in the cells in late apoptosis/dead (2.63%, 13.18% and 1.99% for HOMG W, W/O and control, respectively). On the other hand, percentages not greater than 2.34% were observed in the group of necrotic cells for all treated and control cells. Finally, no significant differences were observed between the heated crude homogenates and control cells. As we observed previously, the exposure of the crude homogenates to high temperatures inhibited their cytotoxic effect. Therefore, the mechanism of cell death of the crude homogenates could be primarily mediated by apoptosis, with a decrease in the cells in the G0/G1 phase and an increase in the cells in the G2/M and subG1 phases, intensified by treatment with the anemone W/O symbiont. Cell cycle analysis of the venom from the sea anemone *Heteractis magnifica* also showed an increase in the cells in the subG1 phase and a decrease in the G1 phase in breast cancer cells (T47D and MCF7) compared to control cells. In addition, this anemone venom also activated caspases-3, -8 and -9, revealing that tumor cell death was dependent on the apoptotic pathway [60]. The same anemone showed cell cycle arrest and cell death by apoptosis in the A549 lung cancer cell line, whereas cell death in non-tumor cells (MRC5) was caused by necrosis [61].

#### 3.6.3. Wound Healing Assay

**Figure 7:**
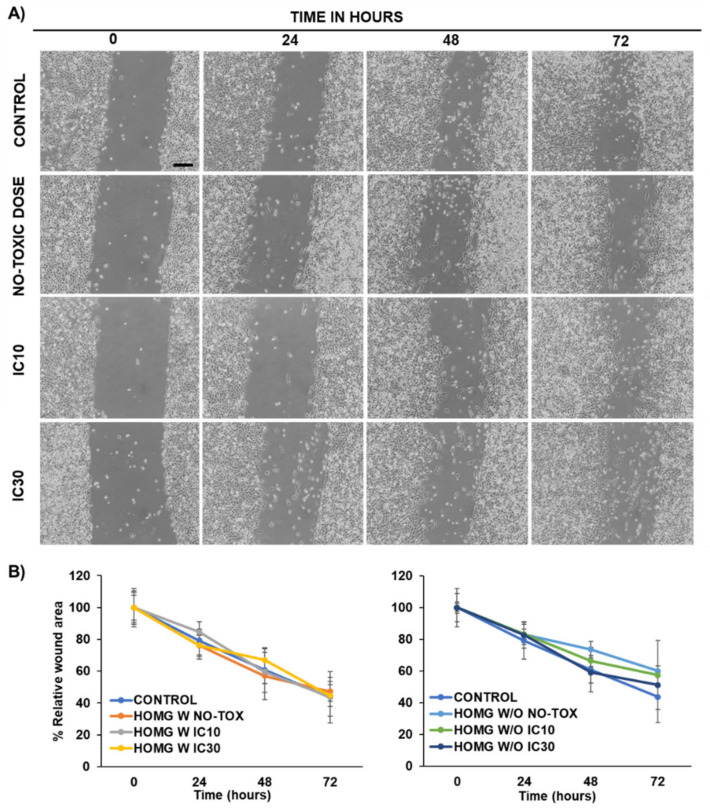
Wound healing assay of the crude homogenates (HOMG) on the T84 human colon cancer cell line. (**A**) Light microscopy images of T84 cells exposed to a non-toxic dose, IC_10_ and IC_30_ of crude HOMG with (W) symbiont for 0 to 72 h after making a wound with a pipette tip. The images were taken at 4× magnification (scale bar = 200 µm). (**B**) Percentage of relative wound area of T84 cells exposed to the crude HOMG W and HOMG W/O relative to the time 0 h (100%). Data are represented as the mean ± standard deviation of triplicate cultures.

To determine the activity of the homogenates over the migration capacity of the colon cancer cells, a wound healing assay was carried out. As shown in Figure 7A and Figure S5, HOMG both W and W/O did not modify cell migration at any of the tested doses. Therefore, although our homogenates showed a clear antiproliferative activity against colon cancer cells, they did not affect cell migration, suggesting no effect on the degree of tumor invasiveness. Some other marine invertebrates contain biological compounds with activity against tumor cell migration. For instance, peptides (CS5931) from the sea squirt *Ciona savignyi* inhibited tumor invasiveness through enolase 1 [62]. In addition, dieckol from *Ecklonia cava*, frondoside A from *Cucumaria frondosa*, geodiamolide H from *Geodia corticostylifera* and pachycladins A–E from *Cladiella pachyclados* demonstrated a similar activity [63]. In contrast, toxin ATX II from *Anemonia Viridis*, enhanced tumor cell migration (9% more) in highly metastatic tumor cells MAT-LyLu but did not modify cell migration in non-metastatic tumor cells (AT-2), as was the case with our cell lines [64]. In this context, more studies are needed to study the modulation of cell migration of our anemone compounds.

#### 3.6.4. Multicellular Tumor Spheroids Antitumor Assay

**Figure 8:**
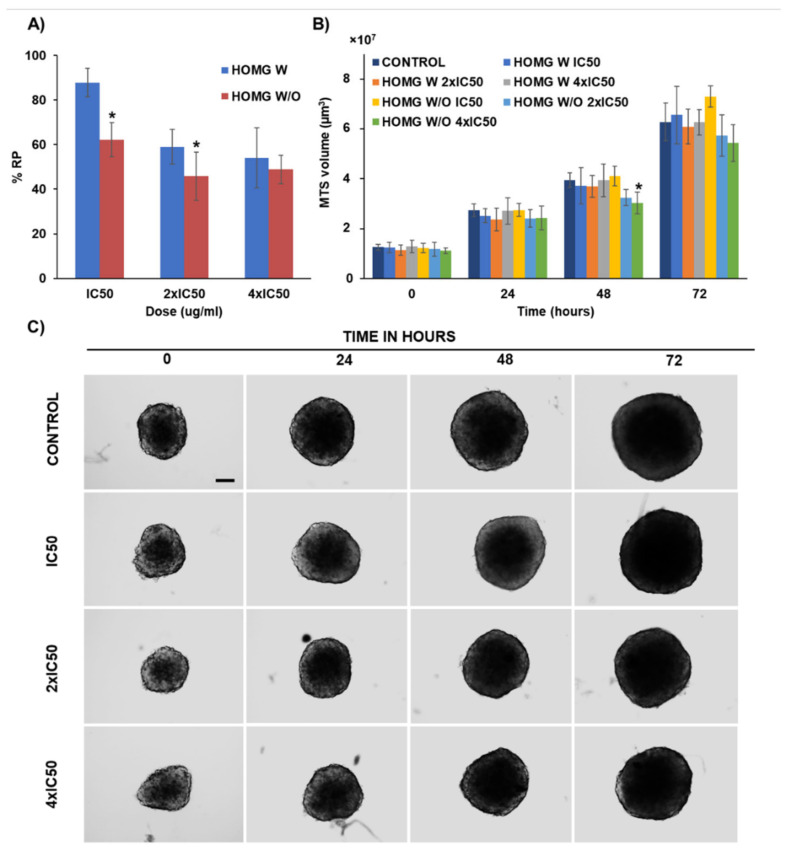
Multicellular tumor spheroids (MTS) from MC38 treated with the crude homogenates (HOMG). (**A**) Proliferation assay with CCK8 on MTS treated for 72 h with HOMG with (W) and without (W/O) symbiont at different doses (IC_50_, 2 × IC_50_ and 4 × IC_50_). (**B**) MTS volume measured at different times of exposure from the beginning of treatment (0, 24, 48 and 72 h). Data are represented as the mean ± standard deviation of octuplicate cultures. (**C**) Light microscopy images of the MTS treated with the crude homogenate without symbiont (HOMG W/O). The images were taken at 10× magnification (scale bar = 100 µm). The symbol * indicates significant differences between crude homogenates with (W) and without (W/O) symbiont.

To determine the effect of our homogenates on a 3D model that mimics primary tumors, we carried out an assay with MTSs which have (as actual in vivo tumors do) a gradient of nutrients, oxygen, etc. [65,66]. As shown in Figure 8A, MTSs from MC38 showed dose-dependent cell death after treatment with both HOMG W and HOMG W/O in comparison with controls. However, HOMG W/O induced a greater volume and proliferation decrease than HOMG W after 72 h of exposure. The most significant differences between both samples were detected at 48 h using a 4 × IC_50_ concentration, and at 72 h MTS became more homogeneous in volume but there were significant differences in the MTS treated with HOMG W/O at IC_50_, 2 × IC_50_ and 4 × IC_50_ (*p* < 0.05) (Figure 8A,C and Figure S6).

## 4. Conclusions

Analysis of the marine invertebrate *Anemonia sulcata* showed a proximate composition with high EPA content, especially in the anemone W/O its symbiont. The simulated digestion of the crude homogenates revealed that more than half of the digested products were potentially dialyzable, although a considerable reduction in their biological properties was detected. Accordingly, the crude homogenates without digestion were tested in cultured cells, in which HOMG W/O demonstrated high antioxidant activity. Furthermore, these crude homogenates also showed an antitumor effect on CRC cells cultured in monolayer and MTS. This antiproliferative effect could be induced by the bleaching process. Therefore, our preliminary results suggest that *Anemonia sulcata* subjected to the bleaching process has a greater antioxidant and antitumor potential than the normal anemone and may be a source of antitumor and antioxidant compounds with nutraceutical applications that could be useful in the treatment and prevention of CRC. Therefore, these extracts could be used in a future as a complement in a diet for CRC chemoprevention, but they should be protected to avoid loss of antioxidant and antitumor thought the digestion process. If the extracts show positive results in vivo, they could be a good complement (orally) for the chemotherapeutic treatment of CRC. In addition, due to their composition in polyunsaturated fatty acids, the consumption of these organisms also provides elements for a healthy diet, which is related to a lower CRC rate. 

## Figures and Tables

**Table 1:** Bioactive compounds (tentative identification) in the crude homogenates from *Anemonia sulcata*.

**HOMG W**									
**RT**	**PPM**	**[M-H]-**	**MF**	**Compound**	**MS Fragments**			**Biological Activity**	**Ref.**
0.98	2	203.056	C_8_H_12_O_6_	* Gadusol	153.0464	1,369,942	119.042	Antioxidant	[41]
1.22	−2.2	315.1225	C_18_H_20_O_5_	Combretastatin A-4	159.0089	151.0379	120.0161	Antitumor	[44,45]
2.73	4	455.2217	C_23_H_37_O_7_P	* 1-Arachidonoylglycerone 3-phosphate (arachidonic acid -ARA derived)	267.0494	119.0465	112.9926	Unknown	[46]
11.8	−2.2	645.3082	C_38_H_46_O_9_	Neogambogic acid	554.2553	553.3141	267.0923	Antitumor	[47]
12.59	−1	599.3214	C_34_H_48_O_9_	Casearupestrin A, B	555.2526	373.0900	313.1264	Antitumor	[48]
14.58	6	603.4449	C_40_H_60_O_4_	* Ophioxanthol (carotenoid)	347.2022	327.2592	301.2049	Antioxidant	[42]
**HOMG W/O**									
**RT**	**PPM**	**[M-H]-**	**MF**	**Compound**	**MS Fragments**			**Biological Activity**	**Ref.**
1.02	0	203.0556	C_8_H_12_O_6_	* Gadusol	173.0172	119.9808	112.9946	Antioxidant	[41]
3.92	−9.5	483.2489	C_32_H_36_O_4_	2-(4-Cyclohexylphenyl)-2-oxoethyl 4-{[4-(2-methyl-2-propanyl)phenoxy] methyl}benzoate	239.0783	119.0519	112.995	Unknown	-
11.44	−0.9	645.3058	C_38_H_46_O_9_	Neogambogic acid	554.2521	433.0973	239.0723	Antitumor	[47]
14.33	−3.7	599.3198	C_34_H_48_O_9_	Casearupestrin A, B	554.2556	415.2043	347.2144	Antitumor	[48]
14.09	−9.9	464.3231	C_29_H_43_N_3_O_2_	Olivoretin A, B, C, E	377.2533	347.2082	267.0982	Inactive	[49]
14.58	2.3	603.4427	C_40_H_60_O_4_	* Ophioxanthol (carotenoid)	348.2070	301.2073	179.0856	Antioxidant	[42]
15.63	3.1	835.5445	C_43_H_80_O_15_	3-{[6-O-(α-D-Galactopyranosyl)-β-D-galactopyranosyl]oxy}-1,2-propanediyl ditetradecanoate	554.255	553.3211	347.2074	Unknown	-

* Compound previously described in marine organisms.

**Table 2:** Total polyphenols content of different samples obtained from *Anemonia sulcata*.

	W Symbiont	W/O Symbiont
HOMG	13.37 ± 0.59	13.77 ± 0.13
EXTOH	12.55 ± 0.19	9.4 ± 0.21
PH	11.23 ± 0.19	9.31 ± 0.52
CONTROL DIAL	5.75 ± 0.45	5.87 ± 0.6
CONTROL RET	4.96 ± 0.27	6.17 ± 0.53
HOMG DIAL	7.26 ± 0.8	9.19 ± 0.95
HOMG RET	6.44 ± 0.3	7.75 ± 0.38

GAE, gallic acid equivalent (GAE). The results are expressed in µg GAE/mg sample as mean ± standard deviation of two samples with four replicates. Crude homogenates (HOMG); ethanolic extracts (EXTOH); protein hydrolysate (PH); dialyzed and retained controls (CONTROL DIAL and CONTROL RET); dialyzed and retained homogenates (HOMG DIAL and HOMG RET); with and without symbiont (W and W/O).

**Table 3:** Free radicals’ uptake/retention of different samples obtained from *Anemonia sulcata*.

	W Symbiont	W/O Symbiont
HOMG	4.71 ± 0.13	5.21 ± 0.04
EXTOH	1.97 ± 0.04	4.4 ± 0.17
PH	9.81 ± 0.9	4.53 ± 0.2
CONTROL DIAL	7.73 ± 0.2	8.66 ± 0.42
CONTROL RET	4.08 ± 0.43	6.59 ± 0.69
HOMG DIAL	0 ± 0.51	1.52 ± 0.5
HOMG RET	1.81 ± 0.11	0.83 ± 0.14

GAE, gallic acid equivalent (GAE). The results are expressed in µg GAE/mg sample as mean ± standard deviation of three replicates. Crude homogenates (HOMG); ethanolic extracts (EXTOH); protein hydrolysate (PH); dialyzed and retained controls (CONTROL DIAL and CONTROL RET); dialyzed and retained homogenates (HOMG DIAL and HOMG RET); with and without symbiont (W and W/O).

## Data Availability

Not applicable.

## Supplementary Materials

The following are available online at https://www.mdpi.com/2079-7737/10/2/134/s1, Figure S1. Antiproliferative activity of the ethanolic extracts in colon cells. Figure S2. Antiproliferative activity of the protein hydrolysates on colon cells. Figure S3. Effect of high temperatures and freeze–thaw cycles on the antiproliferative activity of the crude homogenates (HOMG) on the T84 human colon cancer cell line. Figure S4. Antiproliferative activity of the digestion products from the crude homogenates on the T84 cell line. Figure S5. Wound healing assay of the crude homogenates (HOMG) on the T84 human colon cancer cell line. Figure S6. Multicellular tumor spheroids (MTS) from MC38 treated with the crude homogenates. Table S1. IC_50_ value of colon cells treated with the ethanolic extracts (EXTOH) obtained from *Anemonia sulcata* with (W) and without (W/O) its symbiont.

## Abbreviations

W/O	without
CRC	colorectal cancer
MNPs	marine natural products
MDR	multidrug resistance
HMEC	Human Microvascular Endothelial Cells
MTT	3-(4,5-Dimethylthiazol-2-yl)-2,5-Diphenyltetrazolium Bromide
DMSO	dimethyl sulfoxide
ABTS	2,2′-Azino-bis(3-ethylbenzothiazoline-6-sulfonic acid)
CCK-8	Cell Counting Kit-8
ATCC	American Type Culture Collection
CIC-UGR	Centre for Scientific Instrumentation of the University of Granada
DMEM	Dulbecco’s Modified Eagle’s Medium
PH	protein hydrolysate
SDS-PAGE	sodium dodecyl sulfate–polyacrylamide gel electrophoresis
HOMG	crude homogenates
UPLC	ultra-performance liquid chromatography
QTOF	quadrupole-time-of-flight
MS	mass fragments
RT	retention times
GAE	gallic acid equivalent
%RP	relative proliferation
ETOH	ethanolic extracts
IC_50_	half-maximal inhibitory concentration
MTS	multicellular tumor spheroids
SD	standard deviation
PUFA	polyunsaturated fatty acids
SFA	saturated fatty acids
EPA	5,8,11,14,17-eicosapentaenoic acid
DHA	cis-4,7,10,13,16,19-docosahexaenoic acid
MW	molecular weight
DIAL	dialyzed
RET	retained
ROS	reactive oxygen species
FITC	fluorescein
