# Supplementary material for: Anemonia sulcata and Its Symbiont Symbiodinium as a Source of Anti-Tumor and Anti-Oxidant Compounds for Colon Cancer Therapy: A Preliminary In Vitro Study
Source: Biology (Basel). 2021 Feb 8;10(2):134. doi: 10.3390/biology10020134 (PMC7915377; doi:10.3390/biology10020134)
Supplement: Supplementary file 1 [file biology-10-00134-s001.pdf]

biology

MDPI

Supplementary Materials

Anemonia sulcata and Its Symbiont *Symbiodinium* as a Source of Anti-Tumor and Anti-Oxidant Compounds for Colon Cancer Therapy: A Preliminary In Vitro Study

Laura Cabeza [1,2,3], Mercedes Peña [1,2,3], Rosario Martínez [4], Cristina Mesas [1,2,3], Milagros Galisteo [5], Gloria Perazzoli [1,2,3], Jose Prados [1,2,3,*], Jesús M. Porres [4,†] and Consolación Melguizo [1,2,3,†]

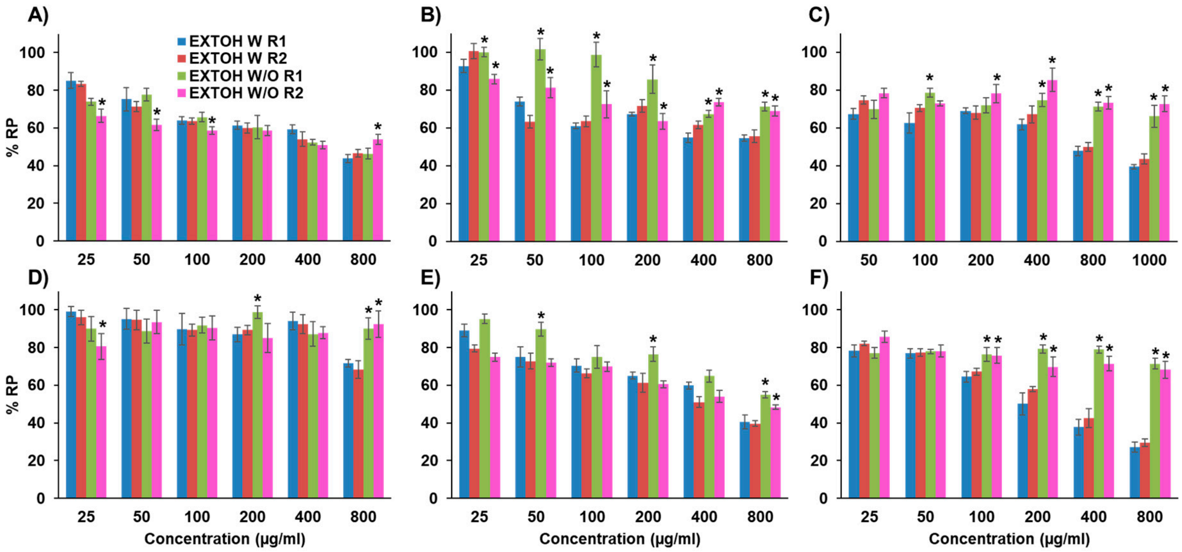

Figure S1. Antiproliferative activity of the ethanolic extracts in colon cells. The T84 (A), HT29 (B), HCT15 (C), SW480 (D) human colon cancer cells, MC38 murine colon cancer cell line (E), and CCD18 non-malignant human colon cell line (F) were exposed to the ethanolic extracts from of *Anemonia sulcata* with (W) and without (W/O) the microalgal symbiont *Symbiodinium* for 72 h from two extractions, replica 1 (R1) and replica 2 (R2). Relative proliferation is expressed as %RP. Data are represented as the mean ± standard deviation of triplicate cultures. The symbol * indicates significant differences between crude homogenates with (W) and without (W/O) symbiont.

Table S1. IC50 value of colon cells treated with the ethanolic extracts (EXTOH) obtained from *Anemonia sulcata* with (W) and without (W/O) its symbiont.

IC50	T84	HT29	HCT15	SW480	MC38	CCD18
**EXTOH W**	545 ± 22.7	805.1 ± 386.6	784.3 ± 144.3	-	473.7 ± 94.3	238.5 ± 42.1
**EXTOH W/O**	870.2 ± 450.9	-	-	-	862.8 ± 246.4	-

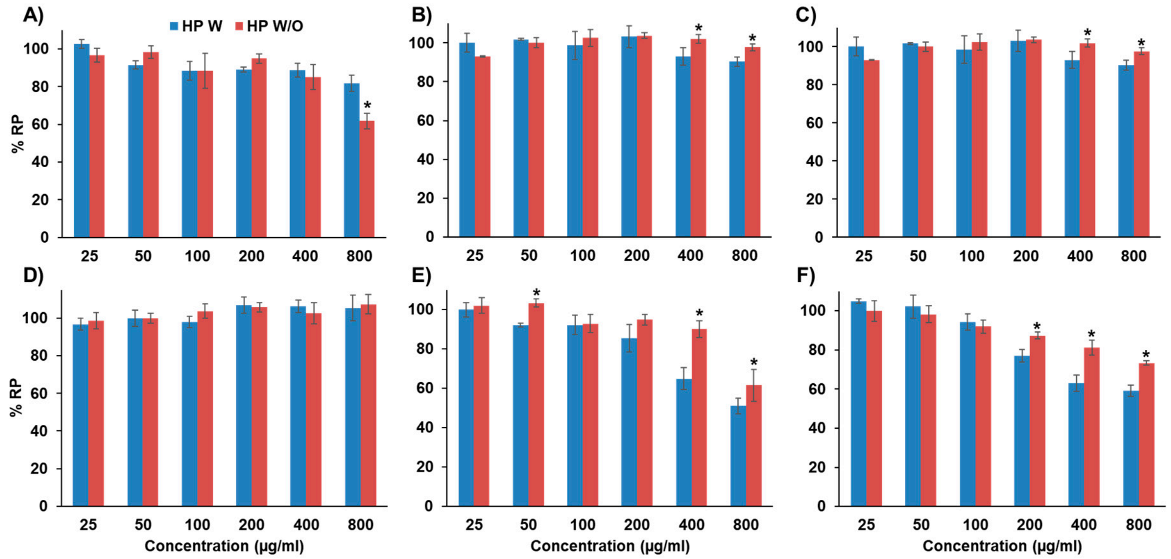

Figure S2. Antiproliferative activity of the protein hydrolysates on colon cells. The T84 (A), HT29 (B), HCT15 (C), SW480 (D) human colon cancer cells, MC38 murine colon cancer cell line (E), and CCD18 non-malignant human colon cell line (F) were exposed to the protein hydrolysates from *Anemonia sulcata* with (W) and without (W/O) its microalgal symbiont *Symbiodinium* for 72 h. Relative proliferation is expressed as %RP. Data are represented as the mean ± standard deviation of triplicate cultures. The symbol * indicates significant differences between crude homogenates with (W) and without (W/O) symbiont.

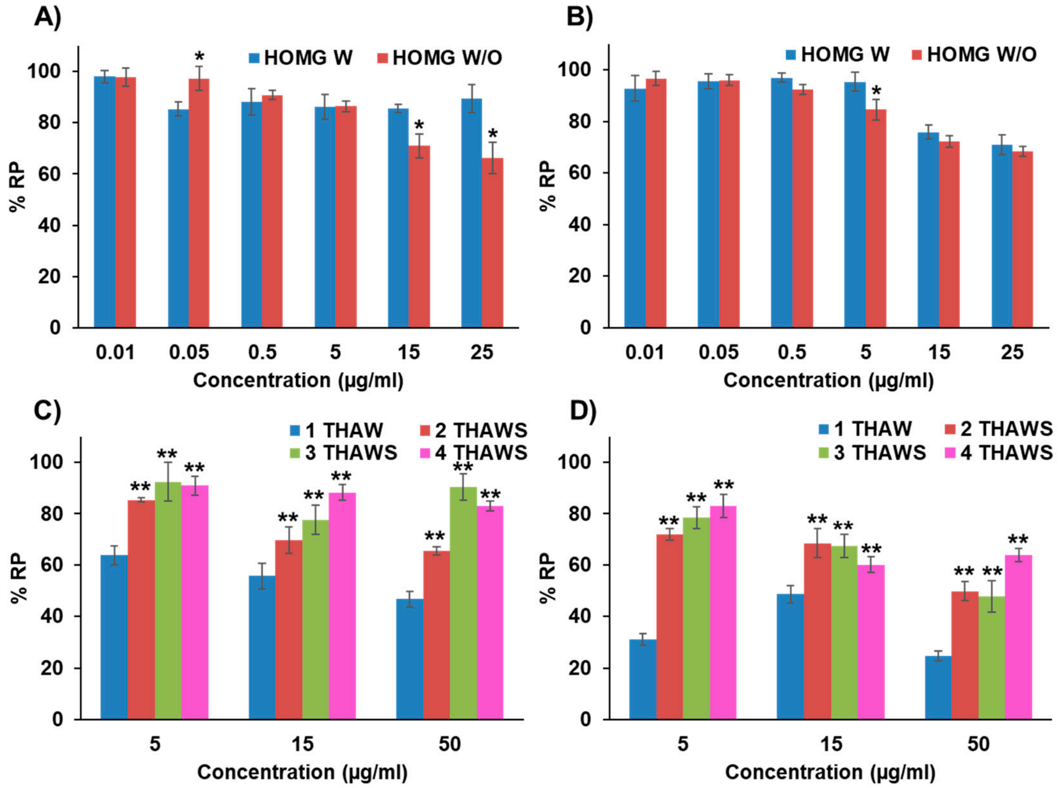

Figure S3. Effect of high temperatures and freeze/thaw cycles on the antiproliferative activity of the crude homogenates (HOMG) on the T84 human colon cancer cell line. (A) HOMG exposed to 96ºC for 5 min in a thermoblock. (B) HOMG exposed to 56ºC for 30 min in a water bath. (C) Cells treated with the crude homogenates with symbiont (W) and (D) without symbiont (W/O) after 1 to 4 freeze and thaw cycles. Relative proliferation is expressed as %RP. Data are represented as the mean ± standard deviation of triplicate cultures. The symbol * indicates significant differences between crude homogenates with (W) and without (W/O) symbiont, and ** indicates significant differences with the first thaw.

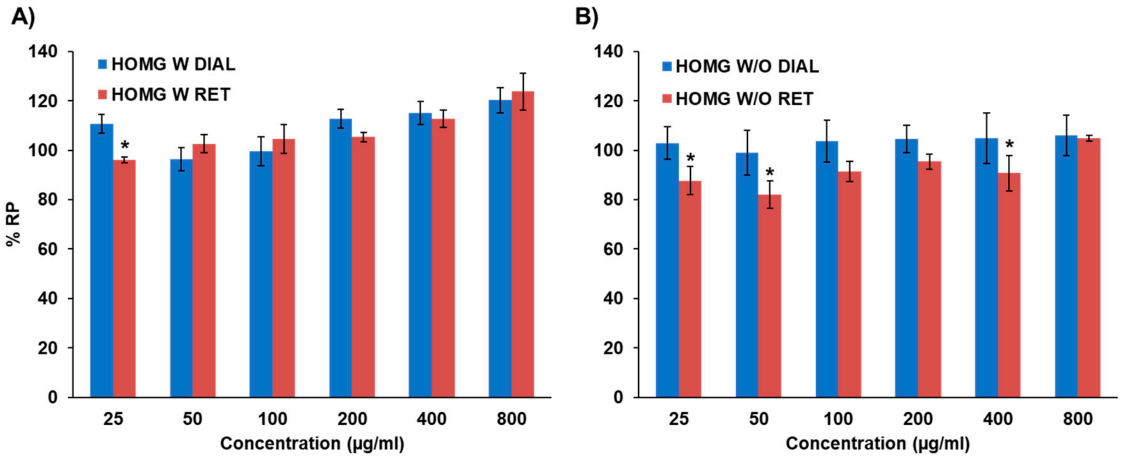

Figure S4. Antiproliferative activity of the digestion products from the crude homogenates on the T84 cell line. Dialyzed (DIAL) and retained (RET) products from digestion of the crude homogenates of *Anemonia sulcata* (A) with (W) and (B) without (W/O) its symbiont *Symbiodinium* for 72 h. Relative proliferation is expressed as %RP and was calculated from control cells and control digestion products. Data are represented as the mean ± standard deviation of triplicate cultures. The symbol * indicates significant differences between DIAL and RET products from digestion of the crude homogenates.

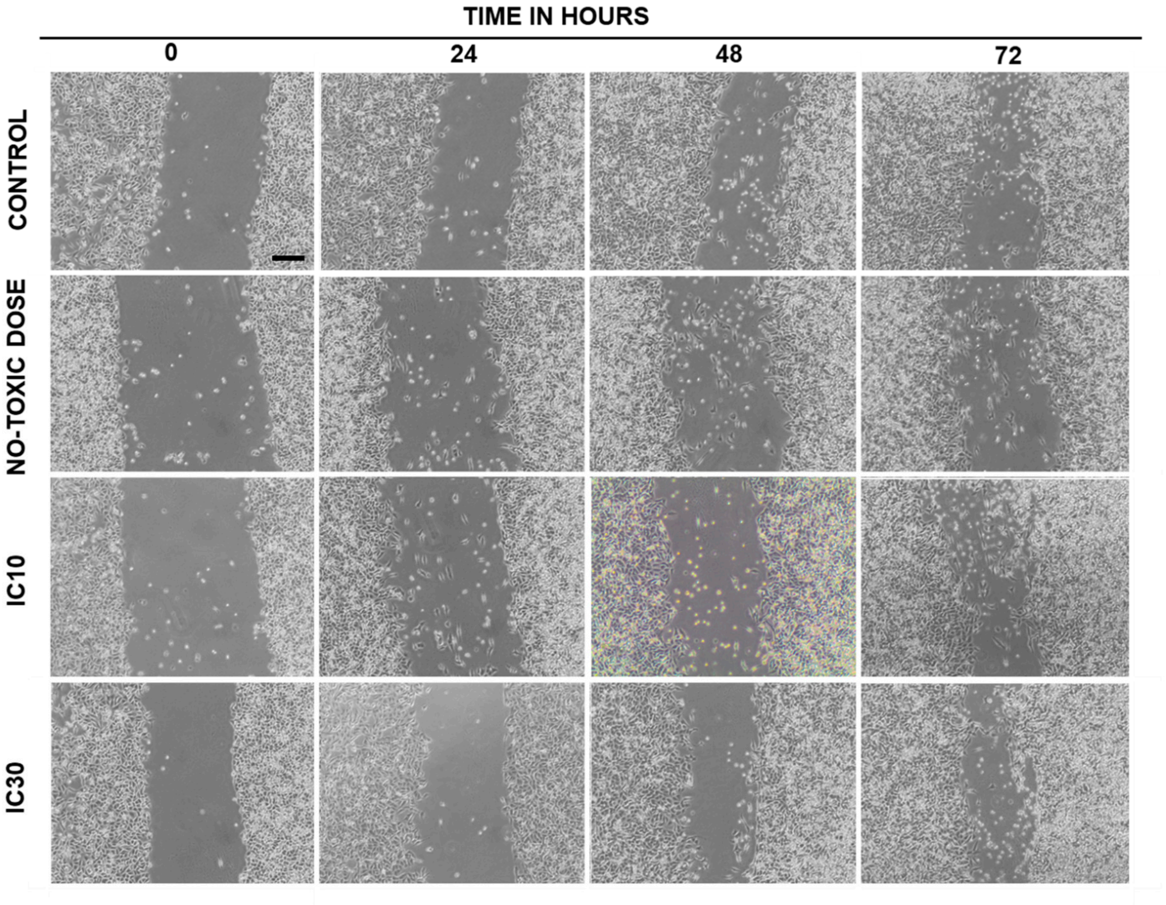

Figure S5. Wound healing assay of the crude homogenates (HOMG) on the T84 human colon cancer cell line. Light microscopy images of T84 cells exposed to a non-toxic dose, IC10 and IC30 of crude HOMG without (W/O) symbiont for 0 to 72 h after making a wound with a pipette tip. The images were taken at 4× magnification (scale bar = 200 µm).

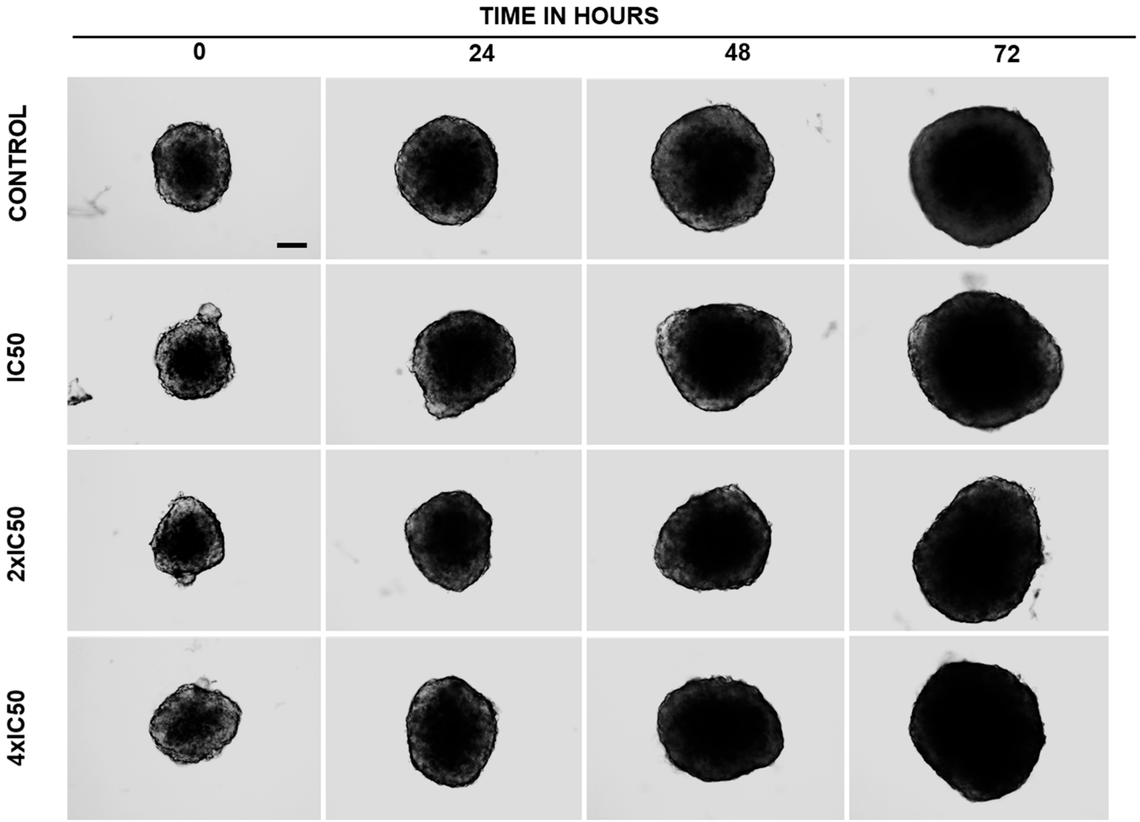

Figure S6. Multicellular tumor spheroids (MTS) from MC38 treated with the crude homogenates. Light microscopy images of the MTS treated with the crude homogenate with symbiont (HOMG W). The images were taken at 10× magnification (scale bar = 100 μm).
